# Hippocampal replay and cortical slow oscillations: a computational study

**DOI:** 10.1186/1471-2202-15-S1-P218

**Published:** 2014-07-21

**Authors:** Paola Malerba, Giri P Krishnan, Maxim Bazhenov

**Affiliations:** 1 Cell Biology and Neuroscience, University of California Riverside, Riverside, CA 92521, USA

## 

Sleep is known to be important for memory consolidation [1], and memories are thought to be stored in the hippocampus during the wakefulness and “transferred” to cortex during sleep [2]. Recently, memory replay – repeatable sequences of pyramidal cell firing – has been demonstrated during sleep, and associated with characteristic brain oscillations, giving rise to the hypothesis that these may form the critical neural substrate of memory consolidation. Tampering with replay can disrupt memory formation and consolidation [3], and the mechanisms underlying sequence replay are still unknown.

During sleep, replay events are associated with specific patterns of neuronal activity. Replay is seen in cortex during sleep oscillation – a rhythmic (< 1Hz) state in which periods of activity (active or Up states) alternate with quiet periods (silent or Down states), while replay in the hippocampus is associated with sharp-wave ripple events – irregularly brief bouts of high frequency (>150 Hz) firing [4]. It is believed that hippocampal ripples may contribute to the cortical active state generation, however specific interactions between ripples and slow oscillation remain unknown.

In the present study, build on our previous research [5], we develop a model of ripple generation to explore interaction between cortical slow oscillation and hippocampal ripples during sleep. Data suggest that during ripple events only a few pyramidal (excitatory) cells are recruited in CA1, and they spike at the peak of the event, while perisomatic interneurons (a kind of inhibitory cells) spike across the duration of the event [6]. The distribution of spike timing with respect to ripple peak is different for different types of interneurons. In hippocampal area CA1, axo-axonic cells tend to spike at ripple initiation and be suppressed in the later part of a ripple [7]. Since axo-axonic cells are in a crucial position to suppress the spikes of the pyramidal cell they impinge upon, we hypothesize that they can regulate the initiation of specific cell activity replay.

In this study we designed a computational model of ripple generation that emphasizes the role of axo-axonic cells in selecting which pyramidal cells are participating in ripple activity, hence what sequence is replayed during a given ripple. In our model, high-frequency firing of the perisomatic interneurons mediate high-frequency LFP oscillations in pyramidal neurons while axo-axonic interneurons define specific sequence of pyramidal cell firing. Using such model, we investigate the timing relationship between cortical slow-wave oscillations and hippocampal ripples. Our study proposes a novel mechanism of hippocampal ripple generation and predicts how interactions between different electrographic events during sleep may contribute to coordinated replay in cortical and hippocampal networks.
